# Mangroves reduce the vulnerability of coral reef fisheries to habitat degradation

**DOI:** 10.1371/journal.pbio.3000510

**Published:** 2019-11-12

**Authors:** Alice Rogers, Peter J. Mumby

**Affiliations:** 1 School of Biological Sciences, Victoria University of Wellington, Wellington, New Zealand; 2 Marine Spatial Ecology Lab and Australian Research Council Centre of Excellence for Coral Reef Studies, School of Biological Sciences, The University of Queensland, Brisbane, Queensland, Australia; University of California, UNITED STATES

## Abstract

Despite general and wide-ranging negative effects of coral reef degradation on reef communities, hope might exist for reef-associated predators that use nursery habitats. When reef structural complexity is lost, refuge density declines and prey vulnerability increases. Here, we explore whether the presence of nursery habitats can promote high predator productivity on degraded reefs by mitigating the costs of increased vulnerability in early life, whilst allowing for the benefits of increased food availability in adulthood. We apply size-based ecosystem models of coral reefs with high and low structural complexity to predict fish biomass and productivity in the presence and absence of mangrove nurseries. Our scenarios allow us to elucidate the interacting effects of refuge availability and ontogenetic habitat shifts for fisheries productivity. We find that low complexity, degraded reefs with nurseries can support fisheries productivity that is equal to or greater than that in complex reefs that lack nurseries. We compare and validate model predictions with field data from Belize. Our results should inform reef fisheries management strategies and protected areas now and into the future.

## Abstract 2

Despite wide-ranging negative effects of coral reef degradation on reef communities, hope might exist for reef-associated predators that use nursery habitats. This study uses size-based ecosystem models of coral reefs to assess the effects of the presence and absence of mangrove nurseries.

## Introduction

As the human population continues to grow, the food security and livelihoods of millions of people in tropical coastal communities depends on the continued productivity of coastal fisheries. For many, coral reef fish provide a major source of dietary protein, as well as livelihoods, a way of life, and cultural identity [1,2]. Yet, climate change and local anthropogenic pressures are causing varying degrees of degradation to coral reefs [3–6]. An important concern is that degraded reefs offer poorer habitat quality [7,8], which is expected to reduce the productivity of future reef fisheries several-fold [9–11].

While loss of reef habitat quality is a significant concern, it is not the only driver of reef fish productivity. The presence of nursery habitats, such as mangroves and seagrass beds, can enhance the biomass of multiple fish species [12–15]. They do this by offering refuge from the high predation associated with coral reefs, while also providing food for rapid growth [16–19]. The mechanism by which nursery habitats influence reef fish dynamics has particular relevance in the context of reef habitat degradation.

The survival of recruiting fish to coral habitats is contingent on their ability to find refuges from predation, typically in crevices and branching corals [20]. Some predators, such as moray eels, have evolved specialised hunting strategies to make use of high structural complexity, hiding within cracks to ambush their prey [21,22]. Many others, however, especially those that rely on speed and vision to hunt, are far more efficient at foraging in locations with low structure [23–25]. Grunts, for example, hunt their prey in rubble beds and seagrass [26], while squirrelfishes emerge from the reef framework at nightfall to hunt on sand [27]. A clear trade-off exists, then, for predatory species: complexity offers protection from one’s own predators but hinders hunting efficiency. Given that most reef fish increase their body size several orders of magnitude throughout ontogeny [28], it necessarily follows that the cost–benefit trade-off of structural complexity for predators varies throughout ontogeny.

In early life, complexity is beneficial and promotes survival when predation risk is high [29]. But as predators grow and move up the food chain, they have fewer predators of their own, and require more food, resulting in a cost of high complexity associated with reduced prey availability. There exists, then, the potential for an important interaction between nursery-habitat use and coral reef habitat quality. If predatory species reduce their post-settlement mortality by using nurseries, a greater flux of larger predators will occupy the reef and take advantage of low habitat quality to forage efficiently. Such fishes will have circumvented the high risk of predation associated with recruiting directly to reefs of low habitat quality. Nursery habitat use might therefore buffer the effects of low habitat quality on the productivity of predatory species. When such productivity is utilised by fisheries, the resulting patterns in fish biomass are likely to be complex. For example, when larger predators are removed through fishing, the predation pressure on smaller predators is reduced, allowing their biomass to accumulate. Such patterns might be most noticeable in reefs of low habitat complexity, where fish biomass is typically low. Here, the presence of both mangrove nurseries and fishing will allow the biomass of unfished predators to rise to levels above those expected in sites lacking nurseries or fishing.

For non-predators, low habitat complexity does not offer the same benefits—food is likely to be equally or indeed less available and abundant when structure is lost. Nursery habitat use will still reduce post-settlement mortality, but if the cost of increased predator abundance on the reef outweigh this benefit, then nurseries might not promote productivity and could potentially have a negative impact on survival for the guild as a whole. Additionally, any negative effect is likely to be higher when habitat quality and therefore refuge availability is low.

Here, we explore the interaction between nursery use and reef habitat quality for predator, herbivore, and total fisheries productivity in the Caribbean. Specifically, we ask whether mangrove nurseries can buffer losses of fisheries productivity as reef habitats decline. To do this, we combine empirical data on nursery habitat use with a field-tested ecosystem model that explicitly captures habitat complexity [9,30,31].

## Results and discussion

**Fig 1:**
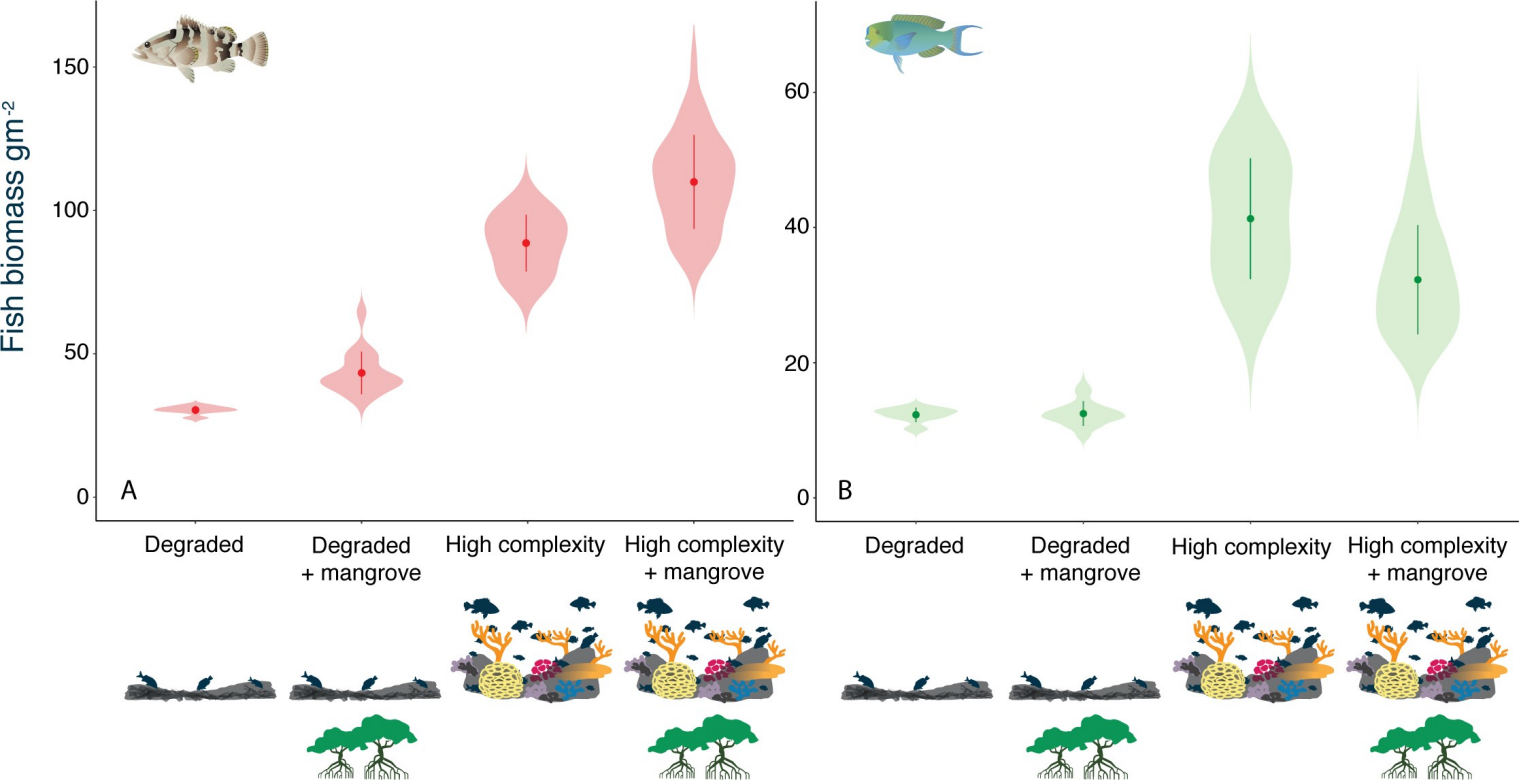
Mangrove effects on biomass. Model predictions showing (A) the slight positive effect of mangrove presence on predator biomass in both degraded and healthy reefs and (B) the slight negative effect of mangrove presence on herbivore biomass in both healthy and degraded reefs. Violin plots show the kernel probability density of the data at different values. Points represent the mean of the data, and bars are equal to one standard error. Plots furthermore confirm the strong positive influence of habitat quality on biomass in both groups. The data underlying this figure can be found in model outputs at https://zenodo.org/badge/latestdoi/185491459.

Models predict that mangroves have positive effects on the biomass of predators (43% increase in low complexity, 24% in high complexity) and negligible or negative effects on the biomass of herbivores (no change in low complexity, 22% decrease in high complexity) (Fig 1). Negative effects on herbivores can be attributed to the positive effects on their predators, which are, on average, more abundant in mangroves than herbivores (0.96 predators m^−2^, versus 0.10 herbivores m^−2^). Predictions are consistent with published empirical studies that utilised 131 reef sites across the Caribbean, and showed that nurseries enhanced the abundance of nursery-using herbivores but reduced that of non–nursery-using species and the assemblage overall [12].

**Fig 2:**
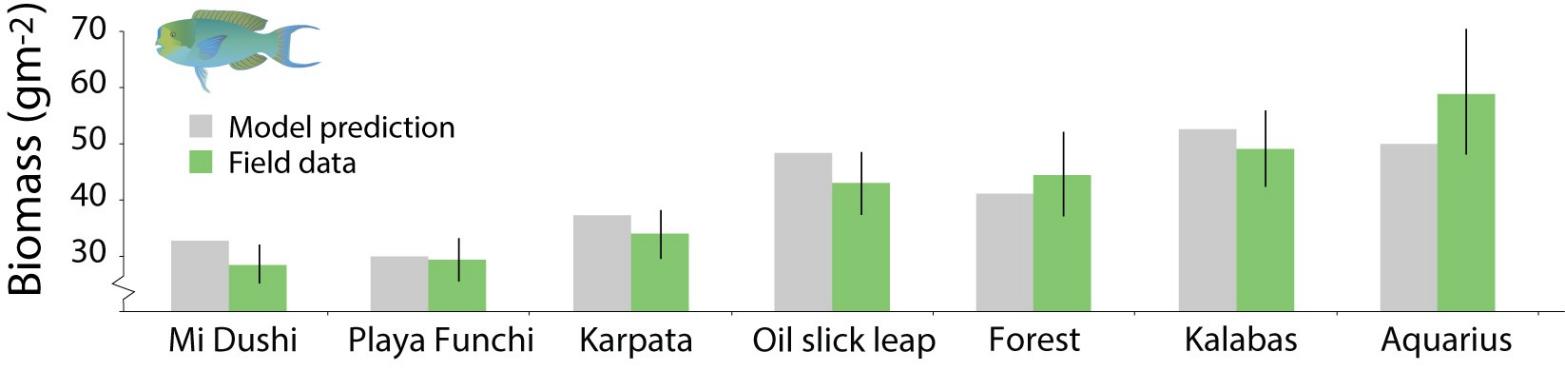
Model validation for Bonaire. Predicted and observed herbivorous fish biomass at seven reef locations in Bonaire varying in habitat structural complexity/refuge availability but lacking mangrove effects. The data underlying this figure can be found in model outputs at https://zenodo.org/badge/latestdoi/185491459.

Our models predict that the biomass of both predators and herbivores responds more strongly to habitat complexity than to nursery availability (Fig 1; 60%–65% increase in predators with high complexity, 61%–70% increase in herbivores). Indeed, our model has been validated by comparing its fit to observed data on parrotfish biomass from the southern Caribbean island of Bonaire. While Bonaire has a small area of mangrove on its southeastern coast, it is too isolated to impact fish assemblages strongly [32], and so comparisons here are limited to scenarios lacking nursery habitats. We used observed size distributions of reef crevices (habitat complexity) to parameterise the model for seven Bonaire locations. Holding all other variables constant, the model reliably captured the strong influence of habitat complexity on the equilibrial biomass of the herbivore assemblage in unfished reefs (Fig 2).

**Fig 3:**
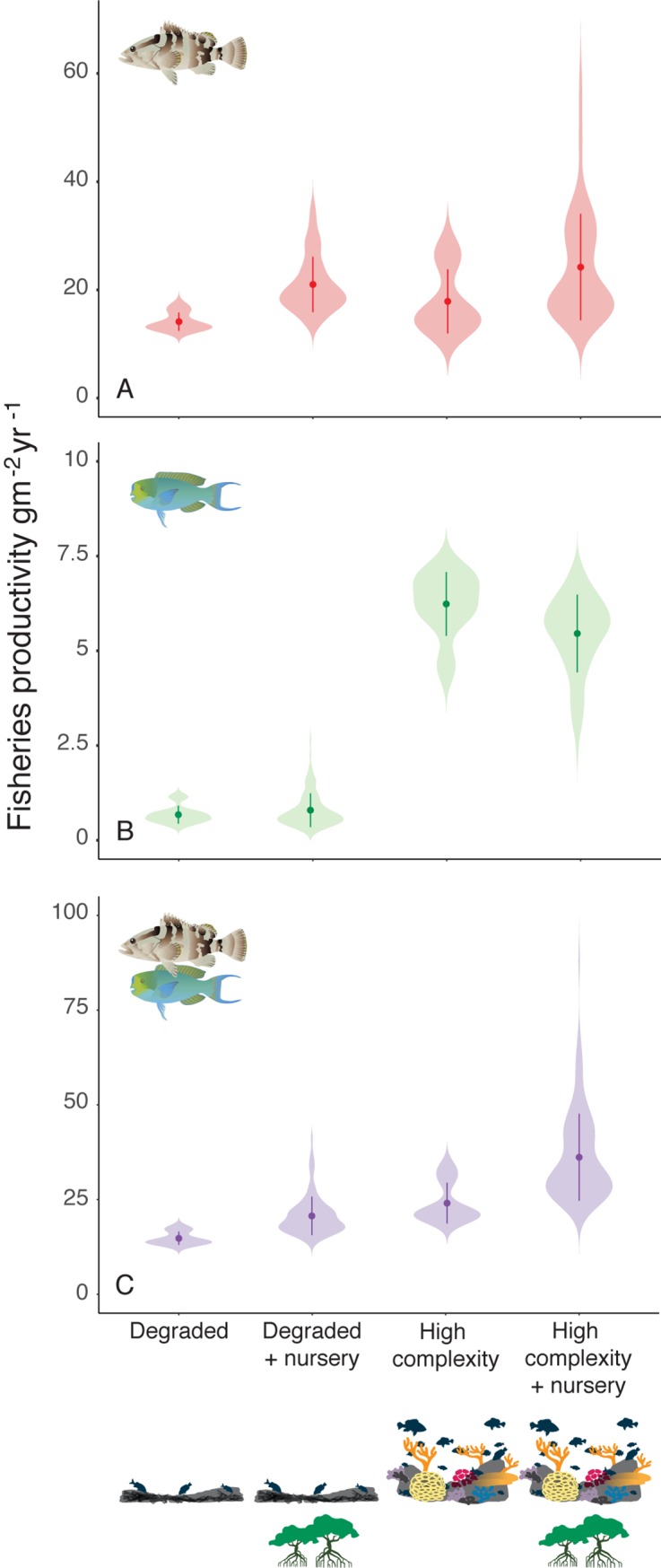
Mangrove effects on the fisheries productivity in high and low complexity reefs. Plots show model predictions for the fisheries productivity of (A) predators, (B) herbivores, and (C) the combined assemblage in scenarios with high and low complexity reefs with and without mangrove nursery supplements. Violin plots show the kernel probability density of the data at different values. Points represent the mean of the data and bars are equal to one standard error. The data underlying this figure can be found in model outputs at https://zenodo.org/badge/latestdoi/185491459.

Fisheries productivity is defined here as the product of growth and abundance for fish of a size range targeted by fisheries. Our models predict that for predators, fisheries productivity increased in the presence of mangrove nurseries. However, a striking result is that mangrove access more than offsets the deleterious impacts of habitat degradation on fisheries productivity for this guild. In other words, the increased productivity resulting from nursery availability is such that degraded reefs with mangroves have equal or greater productivity than healthy reefs without (Fig 3A). For herbivores, however, mangrove nurseries have a slightly negative impact on productivity due to increased predation pressure from predators (Fig 3B). Nevertheless, when predators and herbivores are combined, the positive effects of mangrove nurseries outweigh the costs, and low complexity reefs with mangrove nurseries have similar levels of productivity to high complexity reefs without, supporting the theory that nursery habitats buffer the impacts of habitat degradation on reef fisheries productivity.

The predicted impact of nurseries on productivity is greater than their impact on biomass, for which patterns are mostly driven by habitat complexity. Not surprisingly, the apparent disparity occurs because of growth and turnover. Low complexity habitats with mangrove nurseries have a lower standing stock biomass of predators, but on average, those that are present grow faster in the model (around three times as fast; 305%) due to a greater availability of prey, resulting in higher turnover rates that contribute to productivity. Given that predatory species are primary targets for most coral reef fisheries and generally have a higher market value than herbivorous species, our results suggest that mangrove availability decreases the vulnerability of reef fisheries to habitat degradation and specifically to declines in structural complexity.

**Fig 4:**
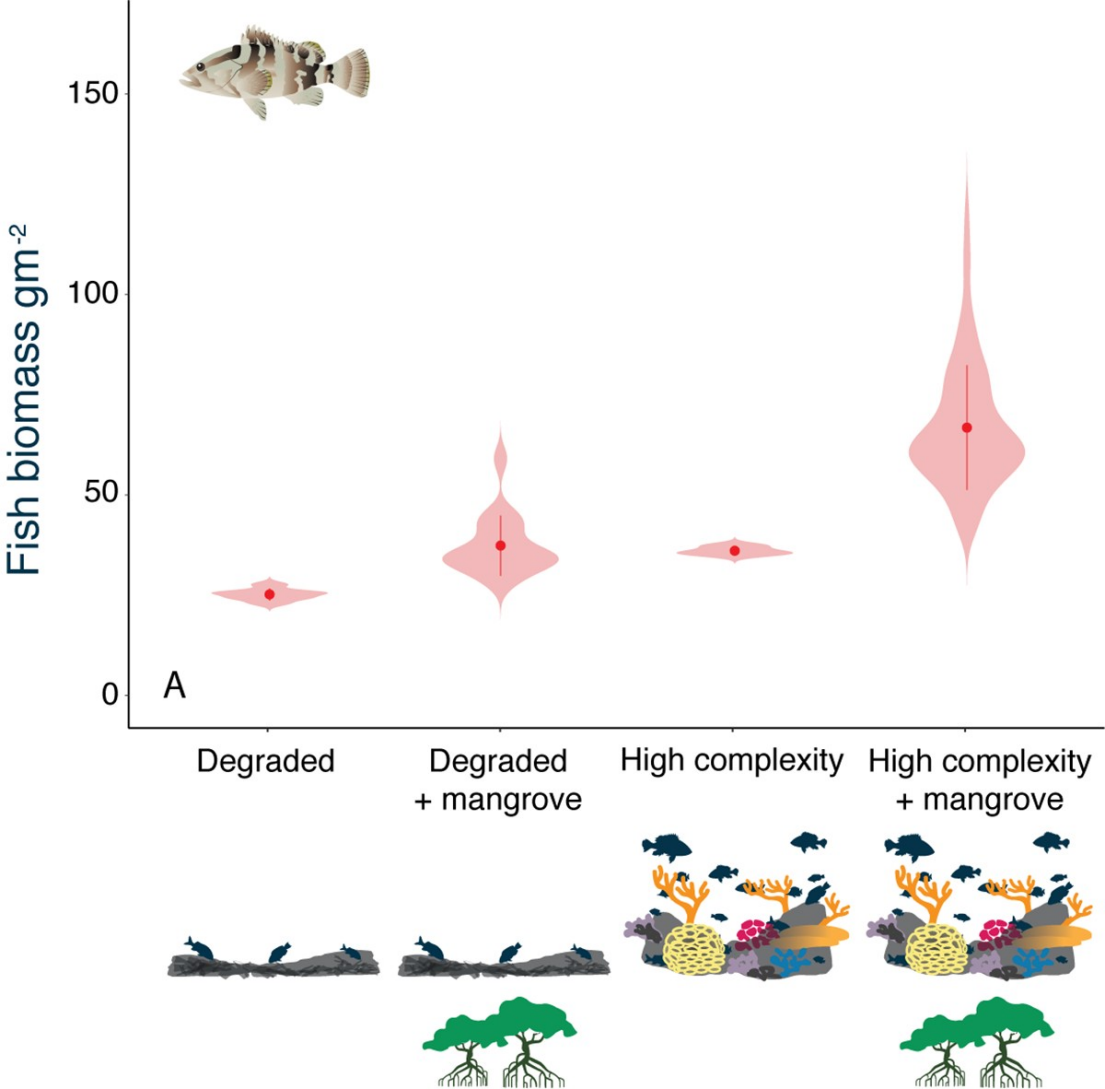
Mangrove effects on biomass under moderate fishing pressure. Reef predator biomass in response to habitat complexity and mangrove nursery availability, and under the influence of moderate (F = 0.4) fishing pressure. The data underlying this figure can be found in model outputs at https://zenodo.org/badge/latestdoi/185491459.

To explore whether fishing modified our observed patterns, we simulated a modest but uniform level of fishing on model systems and examined the consequences for predator fish biomass (Fig 4). The addition of fishing reversed the relative importance of habitat complexity and mangroves on fish biomass. While habitat complexity was the key driver of predator biomass in the absence of fishing (Fig 1), biomass was now greater on reefs with mangrove nurseries, irrespective of habitat complexity (Fig 4). To understand why this is the case, we have to consider the difference between the nonequilibrium conditions in an exploited system, where fishing removes some of the predator biomass, and the equilibrial behaviour of the model without fishing. When the model reaches equilibrium, any additional biomass emanating from mangrove nurseries that is unable to find refuges—as is common in degraded reefs—will be consumed by larger predators and promote productivity without increasing biomass. The removal of some larger would-be predators by fishing means that a proportion of new immigrants in low complexity habitats remain as biomass rather than being consumed and contributing to productivity.

**Fig 5:**
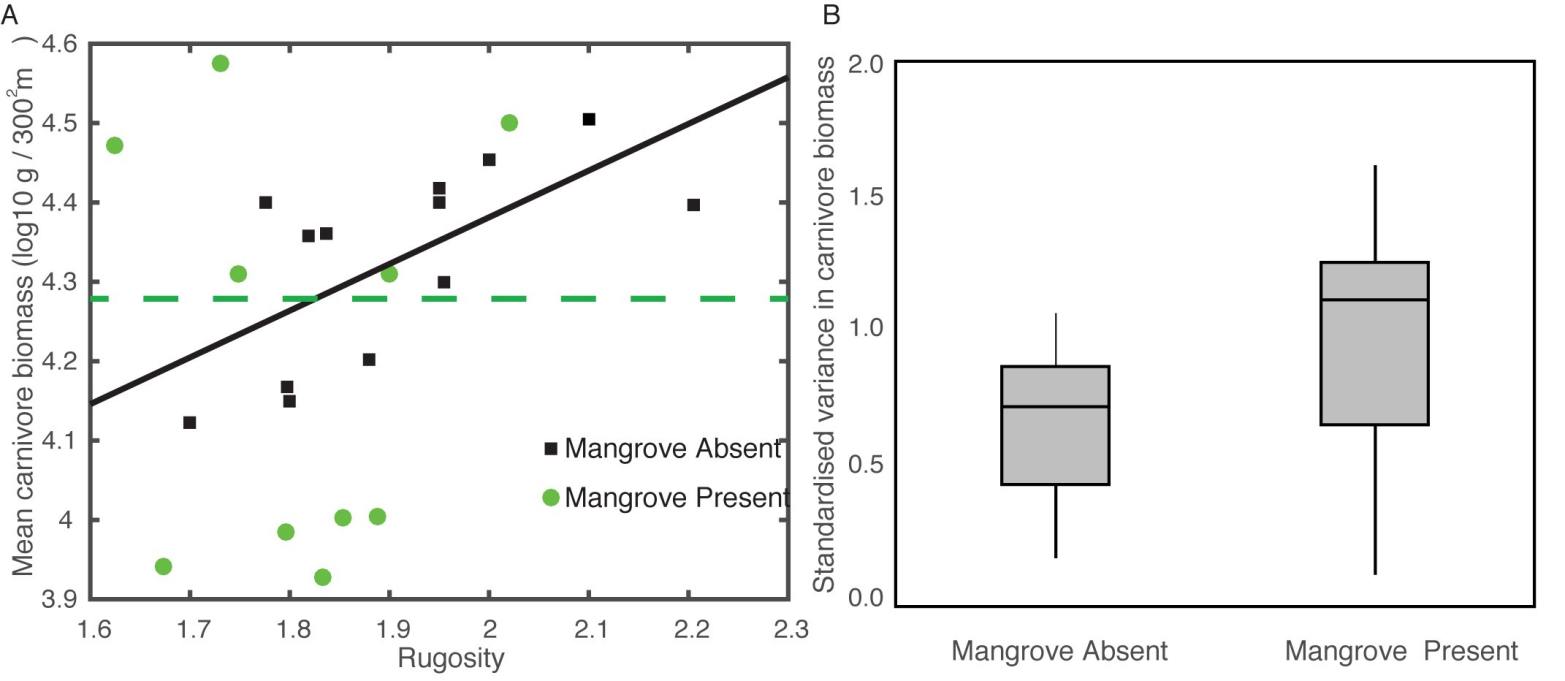
Empirical trends in predator biomass with and without mangrove nurseries. The relationship with habitat complexity (A) and differences in the variance in biomass among sites within a mangrove treatment (B). Panel (A) includes regression fits, where the significant regression for mangroves absent is predator biomass, (log10) = 3.20 + 0.589 * rugosity. The reef site protected from fishing is shown with an open square. Panel (B) presents boxplots of variance in carnivore biomass with median and interquartile range. The data underlying this figure can be found at https://zenodo.org/badge/latestdoi/185491459.

The model also predicts that the positive impact of rugosity on fish biomass will tend to weaken, albeit remain, when the system is fished (Fig 1 vs. Fig 4). We sought empirical support for this model prediction from a field study in Belize that contrasted fish assemblages with and without access to mangrove nurseries [14]. The study sampled at least three reefs within each of six regions, half of which had strong mangrove connectivity and half that lacked mangroves. While the field study did not sample the range of habitat quality considered in the model (it considered moderate to high complexity only), mangroves significantly influenced the nature of the relationship between habitat complexity (rugosity) and predator biomass. In areas rich in mangrove, there was no relationship between carnivore biomass and rugosity (Fig 5A; linear mixed effects model with region as a random effect, *p* = 0.95). Although the overall biomass of carnivores was similar in the sites lacking mangroves (linear mixed effects model, *p* = 0.30), the relationship with rugosity was positive, explaining 30% of variance (Fig 5A; *p* = 0.012). Importantly, fishing pressure was lower in these regions because fishers tend to use mangrove islands for temporary camps and shelter and therefore create greater pressure in the mangrove-rich reefs [14]. Thus, the presence of a positive relationship between rugosity and carnivore biomass is expected in relatively lightly fished systems, even if they lack mangroves (see Fig 4). Note that the only protected reef site in the data set was an area lacking mangroves, which not surprisingly had one of the highest overall biomasses of predators (Fig 4A).

The absence of a relationship between rugosity and predator biomass in mangrove-rich areas is consistent with theory. This is because (1) predators fare relatively well at low rugosity when mangroves allow fishes to circumvent the early predation bottleneck and (2) some of this high productivity at low complexity is manifest as elevated biomass because fishing prevents saturation of predators. That means that a portion of the elevated prey production remains as unconsumed biomass and tends to ‘level out’ the relationship with rugosity. This process of biomass accumulation may be exacerbated by supplemental individuals migrating from mangroves in seasonal bursts and therefore contributing higher biomass that has not yet been consumed and converted into productivity higher up the food chain. We do, however, consider whether variable levels of fishing among mangrove-rich sites could be confounding our interpretation of the absence of a rugosity–biomass relationship. While absolute fishing levels have not been quantified, the variability in fishing intensity is far greater among the six regions (particularly between mangrove-rich and mangrove-poor regions) than among sites sampled within each region, which are easily visited by the same fishers. In contrast, the variability in rugosity occurs among sites within a region; a linear model of rugosity among reefs in the three mangrove-rich regions found no significant differences (*p* = 0.59, *n* = 10). Thus, it is unlikely that the absence of a functional relationship between rugosity and biomass can be attributed to relatively intense fishing at reefs with higher habitat complexity.

Another point of concordance between our model predictions and empirical data can be obtained by comparing the variability of predator biomass between reefs with access to mangrove nurseries versus those without. The model predicts that variance in predator biomass among complex reefs with mangroves is greater than that among complex reefs that lack nurseries (Fig 1; variance without mangroves, 98.1, versus with mangroves, 268.4). This was also borne out in the field data, which were converted to units of standard deviations of the mean in order to compare among sites. The variance among sites in mangrove-rich systems was significantly greater than that among sites lacking mangroves (Fig 5B, Mann-Whitney test of differences in standardised z-scores, which express biomass of carnivores at each site as the number of standard deviations from their shared mean [i.e., their variability], gives *p* = 0.02 with medians of 0.55 versus 1.27 for mangrove-absent versus -rich systems, respectively). Note that there was no significant confounding difference in the rugosity between the mangrove-rich and mangrove-absent treatments (linear model with region nested as a random effect within mangrove category, *p* = 0.16). The enhanced variance among mangrove-rich systems was not associated with rugosity; rather, it reflects the combined effects of variable strength of mangrove-reef nursery habitat connectivity among sites [33] and differences in fishing pressure among regions that prevent some of the biomass gain being converted to productivity. In short, both the field data and models support the conclusion that mangroves reduce the dependency of predators on reef complexity.

## Conclusions

Here, we present both theoretical and empirical support for the conclusion that mangrove nurseries have the capacity to reduce the vulnerability of coral reef fisheries to ongoing and future habitat degradation. While our results offer a glimmer of hope that losses of reef fisheries productivity can be constrained through the provision of a good nursery habitat, this does not undermine the importance of coral reef habitat health nor the impacts of its loss. We predict that biomass on degraded reefs will be lower than on healthy reefs, and this has significant implications for ecosystem functioning and the multitude of services aside from fisheries that coral reefs support [34,35]. Yet, the protection and restoration of mangrove habitats should remain a priority as part of the battle to mitigate climate change impacts on coral reefs and their functioning. Indeed, an interesting avenue for future research would be to consider the design and feasibility of creating artificial mangrove habitat in environments that either do not support natural mangrove forests or have too large a tidal range to provide stable nursery functions in coastal fringes (e.g., subtidal deployment of artificial lagoonal structures).

## Methods

### The model

A size-based, energy-flux ecosystem model for Caribbean coral reefs was used to capture changes in reef fish biomass and productivity under different habitat complexity and nursery scenarios. The model describes production and predation links between three size-structured trophic groups. Predators feed on other predators, herbivores, and invertebrates in a size-based manner, determined by an average predator–prey mass ratio. Herbivores graze turf algae, which is replenished at every time step based upon in situ estimates of benthic productivity. Finally, invertebrates feed on detritus, which fluxes into the system as a result of death and defecation in all three trophic groups.

### Reef complexity parameterisation

Coral reef structural complexity is described in the model by the abundance and size distribution of reef crevices. For model validation, reef crevices were measured in 5-cm size classes in situ at seven reef locations inside the marine reserve on the southern Caribbean island of Bonaire. At each time step in the model, the density of available refuges in a given size class determines what proportion of prey are vulnerable to predation, versus safe in hiding places. Given that refuge density remains constant through time, the shape of the resulting vulnerability function is density dependent and thus implicitly captures competition for refuge space.

To explore how the availability of mangrove nurseries interacts with habitat complexity to determine fisheries productivity, we ran model scenarios based on a factorial design: complex and noncomplex coral reefs with and without nursery habitats. We considered seven complex coral reef scenarios based on the measured crevice data from the seven reef locations in Bonaire for which the model has previously been validated. This allowed us to capture variability in model predictions that is driven by realistic differences in the size distribution of refuges on relatively healthy, complex reefs. Degraded reefs were modelled by removing from the data small refuges between 5 and 30 cm in size, because many of these are provided by branching corals, which are the first to erode in the face of habitat degradation. In addition, the abundance of crevices >30 cm was reduced by 50% to capture the loss of many of the spaces between corals, whilst allowing for the persistence of some larger structures associated with the underlying topography of the substrate. As a result, there were seven low complexity reef scenarios, each representing the simulated degradation of the Bonaire reefs for which the model was validated.

### Mangrove nursery effect parameterisation

**Fig 6:**
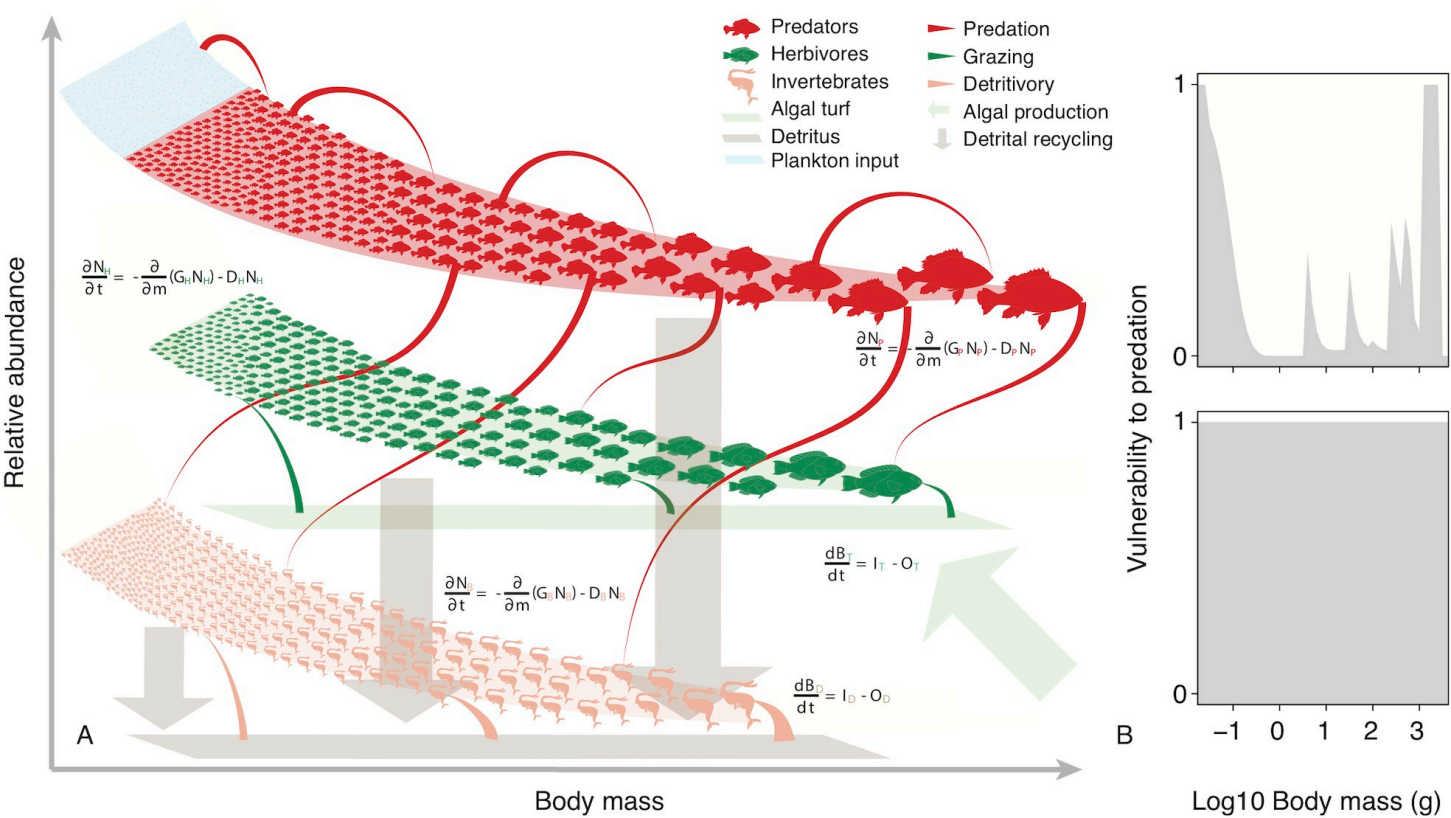
The size-based ecosystem model. (A) Schematic overview of the size-based, energy-flux ecosystem model showing predator, herbivore, and invertebrate size spectra and associated fluxes in primary production and detritus. (B) Examples of the vulnerability function for reefs with high (upper) and zero (lower) habitat complexity.

Fish density data from 16 mangrove habitat sites around four reef locations (Lighthouse North, Tobacco North, Tobacco South, and Turneffe) in Belize (data sourced from [14]) provided estimates for the abundance of reef fish available to migrate to coral reefs if an adjacent mangrove nursery was present. To account for ontogenetic migration, whereby fish remain in nurseries until they are large enough to have improved survival in the adult habitat, we added all supplemental fish (total density across size classes) at a size of approximately 16–18 cm, which is the size at which fish appear to move onto the forereef from nursery habitats [14]. Supplemental individuals were added to the relevant predatory or herbivorous size spectrum in a model run at each time step (representing constant replenishment). We had 14 non-nursery scenarios in which no supplemental fish were added: seven for complex reefs and seven for degraded reefs. In addition, all possible nursery supplements were applied to all possible reef complexities; seven complex, seven degraded, resulting in 224 (16 × 14) nursery scenarios. Based on observed differences between the area covered by mangrove habitat versus coral reef habitat in Belize (approximately 2 times as much mangrove [14]), we doubled the density of fish in mangroves to capture realistic densities, arriving at our modelled reefs. For every model run, biomass and productivity (the product of abundance and growth) of predatory fish was calculated when the model had reached equilibrium. Fig 6 provides a schematic overview of the model (A) and examples of the vulnerability function for reefs with high and no structural complexity (B). Model equations (S1 Table), parameters (S2 Table), and additional model details (S3 and S4 Tables and S1 Text) are provided as supporting information.

### Belize data for model testing and comparison

Model predictions relating to nursery and complexity effects on fish biomass were compared to field surveys from at least three *Orbicella* reefs within each of six regions in Belize. Half of the sites surveyed had strong mangrove connectivity, whilst half lacked mangroves. At each site, fish biomass was quantified using belt transects (see Supporting information for full details). In addition, the chain and tape method was used to obtain a simple metric of reef rugosity. The sites selected for this aspect of the study were not primarily chosen based on their structural complexity, and as a result, all fell within a medium to high complexity range.

## Supporting information

S1 Table Model equations. (DOCX)

S2 Table Parameter definitions, values, and units. (DOCX)

S3 Table Allocation of empirically measured crevice densities to model fish body mass increments based on length–weight conversion parameters; a = 0.025, b = 3, and the relationship, weight = a * length ^ b. (DOCX)

S4 Table Supplemental predator and herbivore fish densities from 16 mangrove sites in Belize. (DOCX)

S1 Text Model descriptions and parameter justification. (DOCX)

## References

[1] Díaz S, Demissew S, Carabias J, Joly C, Lonsdale M, Ash N, et al The IPBES Conceptual Framework—connecting nature and people. Current Opinion in Environmental Sustainability. 2015;14:1–16. 10.1016/j.cosust.2014.11.002.

[2] FAO. The state of world fisheries and aquaculture 2016. Contributing to food security and nutrition for all. Rome: 2016.

[3] Hughes TP, Kerry JT, Alvarez-Noriega M, Alvarez-Romero JG, Anderson KD, Baird AH, et al Global warming and recurrent mass bleaching of corals. Nature. 2017;543(7645):373–7. Epub 2017/03/17. 10.1038/nature21707 . 28300113

[4] Wolff NH, Mumby PJ, Devlin M, Anthony KRN. Vulnerability of the Great Barrier Reef to climate change and local pressures. Glob Chang Biol. 2018;24(5):1978–91. Epub 2018/02/09. 10.1111/gcb.14043 . 29420869

[5] Ortiz JC, Wolff NH, Anthony KRN, Devlin M, Lewis S, Mumby PJ. Imparied recovery of the Great Barrier Reef under cumulative stress. Science Advances. 2018;4:1–8. 10.1126/sciadv.aar6127 PMC6051737 30035217

[6] Perry CT, Alvarez-Filip L, Graham NAJ, Mumby PJ, Wilson SK, Kench PS, et al Loss of coral reef growth capacity to track future increases in sea level. Nature. 2018;558(7710):396–400. 10.1038/s41586-018-0194-z . 29904103

[7] Wilson SK, Babcock RC, Fisher R, Holmes TH, Moore JAY, Thomson DP. Relative and combined effects of habitat and fishing on reef fish communities across a limited fishing gradient at Ningaloo. Marine Environmental Research. 2012;81:1–11. 10.1016/j.marenvres.2012.08.002 22925735

[8] Gratwicke B, Speight MR. Effects of habitat complexity on Caribbean marine fish assemblages. Mar Ecol-Prog Ser. 2005;292:301–10.

[9] Rogers A, Blanchard JL, Mumby PJ. Vulnerability of coral reef fisheries to a loss of structural complexity. Curr Biol. 2014;24(9):1000–5. 10.1016/j.cub.2014.03.026 24746794

[10] Rogers A, Blanchard JL, Mumby PJ. Fisheries productivity under progressive coral reef degradation. J Appl Ecol. 2018;55:1041–9.

[11] Pratchett MS, Hoey AS, Wilson SK. Reef degradation and the loss of critical ecosystem goods and services provided by coral reef fishes. Current Opinion in Environmental Sustainability. 2014;7:37–43. 10.1016/j.cosust.2013.11.022

[12] Harborne AR, Nagelkerken I, Wolff NH, Bozec Y-M, Dorenbosch M, Grol MGG, et al Direct and indirect effects of nursery habitats on coral-reef fish assemblages, grazing pressure and benthic dynamics. Oikos. 2016;125(7):957–67. 10.1111/oik.02602

[13] Nagelkerken I, Roberts CM, van der Velde G, Dorenbosch M, van Riel MC, Cocheret de la Moriniere E, et al. How important are mangroves and seagrass beds for coral-reef fish? The nursery hypothesis tested on an island scale. Mar Ecol-Prog Ser. 2002;244:299–305.

[14] Mumby PJ, Edwards AJ, Ernesto Arias-Gonzalez J, Lindeman KC, Blackwell PG, Gall A, et al Mangroves enhance the biomass of coral reef fish communities in the Caribbean. Nature. 2004;427(6974):533–6. http://www.nature.com/nature/journal/v427/n6974/suppinfo/nature02286_S1.html. 10.1038/nature02286 14765193

[15] Igulu MM, Nagelkerken I, M. D, Grol MGG, Harborne AR, Kimirei IA, et al Mangrove habitat use by juvenile reef fish: Meta-analysis reveals that tidal regime matters more than biogeographic region. PLoS ONE. 2014;9(12):e0114715 10.1371/journal.pone.0114715 25551761 PMC4281128

[16] Kimirei IA, Nagelkerken I, Trommelen M, Blankers P, van Hoytema N, Hoeijmakers D, et al What drives ontogenetic niche shifts of fishes in coral reef ecosystems? Ecosystems. 2013;16(5):783–96. 10.1007/s10021-013-9645-4

[17] Dahlgren CP, Eggleston DB. Ecological processes underlying ontogenetic habitat shifts in a coral reef fish. Ecology. 2000;81(8):2227–40. 10.1890/0012-9658(2000)081[2227:EPUOHS]2.0.CO;2

[18] Laegdsgaard P, Johnson C. Why do juvenile fish utilise mangrove habitats? J Exp Mar Biol Ecol. 2001;257(2):229–53. ISI:000167646800006. 11245878 10.1016/s0022-0981(00)00331-2

[19] Chittaro PM, Usseglio P, Sale P. Variation in fish density, assemblage composition and relative rates of predation among mangrove, seagrass and coral reef habitats. Environmental Biology of Fishes. 2005;72(2):175–87. 10.1007/s10641-004-9077-2 WOS:000227371300007.

[20] Hixon MA, Beets JP. Predation, Prey Refuges, and the Structure of Coral-Reef Fish Assemblages. Ecol Monogr. 1993;63(1):77–101. ISI:A1993MN18500004.

[21] Almany GR. Differential effects of habitat complexity, predators and competitors on abundance of juvenile and adult coral reef fishes. Oecologia. 2004;141(1):105–13. Epub 2004/06/16. 10.1007/s00442-004-1617-0 . 15197644

[22] Feeney WE, Lönnstedt OM, Bosiger Y, Martin J, Jones GP, Rowe RJ, et al High rate of prey consumption in a small predatory fish on coral reefs. Coral Reefs. 2012;31(3):909–18. 10.1007/s00338-012-0894-z

[23] Hixon MA, Carr MH. Synergistic pedation, density dependence, and population regulation in marine fish. Science. 1997;277(5328):946–9. 10.1126/science.277.5328.946

[24] Greenberg LA, Paszkowski CA, Tonn WM. Effects of prey species composition and habitat structure on foraging by two functionally distinct piscivores. Oikos. 1995;74:522–32.

[25] Beukers-Stewart BD, Jones GP. The influence of prey abundance on the feeding ecology of two piscivorous species of coral reef fish. Journal of Experimental Marine Biology and Ecology. 2004;299:155–84. 10.1016/j.jembe.2003.08.015

[26] Lieske E, Myers R. Collins Pocket Guide. Coral reef fishes Indo-Pacific & Caribbean including the Red Sea. Harper Collins Publishers, 400 p. 1994.

[27] Woods LP, Greenfield DW. Holocentridae In Fischer W. (ed). FAO species identification sheets for fishery purposes. Western Central Atlantic (Fishing Area 31). Vol 3, FAO, Rome 1978.

[28] Werner EE, Gilliam JF. The ontogenetic niche and species interactions in size-structured populations. Annual Review of Ecology and Systematics. 1984;15(1):393–425. 10.1146/annurev.es.15.110184.002141

[29] Hixon MA, Anderson TW, Buch KL, Johnson DW, McLeod JB, Stallings CD. Density dependence and population regulation in marine fish: a large-scale, long-term field manipulation. Ecol Monogr. 2012;82(4):467–89. 10.1890/11-1525.1 WOS:000312012400004.

[30] Rogers A, Blanchard JL, Mumby PJ. Fisheries productivity under progressive coral reef degradation. Journal of Applied Ecology. 2017 10.1111/1365-2664.13051

[31] Rogers A, Blanchard JL, Newman SP, Dryden CS, Mumby PJ. High refuge availability on coral reefs increases the vulnerability of reef-associated predators to overexploitation. Ecology. 2018 10.1002/ecy.2103 . 29328509

[32] Nagelkerken I, Huebert KB, Serafy JE, Grol MGG, Dorenbosch M, Bradshaw CJA. Highly localized replenishment of coral reef fish populations near nursery habitats. Marine Ecology Progress Series. 2017;568:137–50. 10.3354/meps12062

[33] Edwards HJ, Elliott IA, Pressey RL, Mumby PJ. Incorporating ontogenetic dispersal, ecological processes and conservation zoning into reserve design. Biol Conserv. 2010;143(2):457–70. 10.1016/j.biocon.2009.11.013 WOS:000274761000021.

[34] Brown CJ, Mumby PJ. Trade-offs between fisheries and the conservation of ecosystem function are defined by management strategy. Front Ecol Environ. 2014;12(6):324–9. 10.1890/130296 WOS:000340083200014.

[35] Rogers A, Harborne AR, Brown CJ, Bozec YM, Castro C, Chollett I, et al Anticipative management for coral reef ecosystem services in the 21st century. Glob Chang Biol. 2015;21(2):504–14. 10.1111/gcb.12725 . 25179273

