# Supplementary material for: Mangroves reduce the vulnerability of coral reef fisheries to habitat degradation
Source: PLoS Biol. 2019 Nov 12;17(11):e3000510. doi: 10.1371/journal.pbio.3000510 (PMC6850520; doi:10.1371/journal.pbio.3000510)
Supplement: S1 Table — (DOCX) [file pbio.3000510.s001.docx]

**S1 Table**: Model equations. Subscripts: *i* (*P* = predatory fish, *H* = herbivorous fish, *B* = benthic detritivores), *D* = detritus and *T* = turf algae.

| Equations | Units |  |
| --- | --- | --- |
| Dynamical system:  $\frac{\boldsymbol{\partial}\boldsymbol{N}_{\boldsymbol{P}}}{\boldsymbol{\partial t}}\boldsymbol{=-}\frac{\boldsymbol{\partial}}{\boldsymbol{\partial m}}\left( \boldsymbol{G}_{\boldsymbol{P}}\boldsymbol{N}_{\boldsymbol{P}} \right)\boldsymbol{-}\boldsymbol{D}_{\boldsymbol{P}}\boldsymbol{N}_{\boldsymbol{P}}$  $\frac{\boldsymbol{\partial}\boldsymbol{N}_{\boldsymbol{H}}}{\boldsymbol{\partial t}}\boldsymbol{=-}\frac{\boldsymbol{\partial}}{\boldsymbol{\partial m}}\left( \boldsymbol{G}_{\boldsymbol{H}}\boldsymbol{N}_{\boldsymbol{H}} \right)\boldsymbol{-}\boldsymbol{D}_{\boldsymbol{H}}\boldsymbol{N}_{\boldsymbol{H}}$  $\frac{\boldsymbol{\partial}\boldsymbol{N}_{\boldsymbol{B}}}{\boldsymbol{\partial t}}\boldsymbol{=-}\frac{\boldsymbol{\partial}}{\boldsymbol{\partial m}}\left( \boldsymbol{G}_{\boldsymbol{B}}\boldsymbol{N}_{\boldsymbol{B}} \right)\boldsymbol{-}\boldsymbol{D}_{\boldsymbol{B}}\boldsymbol{N}_{\boldsymbol{B}}$  $\frac{\boldsymbol{d}\boldsymbol{B}_{\boldsymbol{D}}}{\boldsymbol{dt}}\boldsymbol{=}\boldsymbol{I}_{\boldsymbol{D}}\boldsymbol{-}\boldsymbol{O}_{\boldsymbol{D}}$  $\frac{\boldsymbol{d}\boldsymbol{B}_{\boldsymbol{T}}}{\boldsymbol{dt}}\boldsymbol{=}\boldsymbol{I}_{\boldsymbol{T}}\boldsymbol{-}\boldsymbol{O}_{\boldsymbol{T}}$ | m^-2^ year^-1^ g^-1^  m^-2^ year^-1^ g^-1^  m^-2^ year^-1^ g^-1^  m^-2^ year^-1^ g^-1^  m^-2^ year^-1^ g^-1^ | (M1)  (M2)  (M3)  (M4)  (M5) |
| Flux terms from feeding result from prey preference, allometric search rates and availability across the size spectrum:  $\boldsymbol{F}_{\boldsymbol{Pi}}\left( \boldsymbol{m,t} \right)\boldsymbol{=}\boldsymbol{\omega}_{\boldsymbol{i}}\boldsymbol{A}_{\boldsymbol{P}}\boldsymbol{m}^{\boldsymbol{\alpha}_{\boldsymbol{P}}}\boldsymbol{V}_{\boldsymbol{P}}\int\boldsymbol{\varphi(m/}\boldsymbol{m}^{\boldsymbol{'}}\boldsymbol{)}\boldsymbol{N}_{\boldsymbol{i}}\left( \boldsymbol{m}^{\boldsymbol{'}}\boldsymbol{,t} \right)\boldsymbol{m}^{\boldsymbol{'}}\boldsymbol{d}\boldsymbol{m}^{\boldsymbol{'}}$  $\boldsymbol{F}_{\boldsymbol{H}}\left( \boldsymbol{m,t} \right)\boldsymbol{=}\boldsymbol{A}_{\boldsymbol{H}}\boldsymbol{m}^{\boldsymbol{\alpha}_{\boldsymbol{H}}}\boldsymbol{B}_{\boldsymbol{T}}\boldsymbol{(t)}$  $\boldsymbol{F}_{\boldsymbol{B}}\left( \boldsymbol{m,t} \right)\boldsymbol{=}\boldsymbol{A}_{\boldsymbol{B}}\boldsymbol{m}^{\boldsymbol{\alpha}_{\boldsymbol{B}}}\boldsymbol{B}_{\boldsymbol{D}}\boldsymbol{(t)}$ | g year^-1^ | (M6)  (M7)  (M8) |
| Feeding rates combined with gross growth conversion efficiency give relative growth rates:  $\boldsymbol{G}_{\boldsymbol{P}}\left( \boldsymbol{m,t} \right)\boldsymbol{=}\boldsymbol{K}_{\boldsymbol{P}}\boldsymbol{F}_{\boldsymbol{PP}}\left( \boldsymbol{m,t} \right)\boldsymbol{+}{\boldsymbol{K}_{\boldsymbol{H}}\boldsymbol{F}_{\boldsymbol{PH}}\left( \boldsymbol{m,t} \right)\boldsymbol{+K}}_{\boldsymbol{B}}\boldsymbol{F}_{\boldsymbol{PB}}\boldsymbol{(m,t)}$  $\boldsymbol{G}_{\boldsymbol{H}}\left( \boldsymbol{m,t} \right)\boldsymbol{=}\boldsymbol{K}_{\boldsymbol{T}}\boldsymbol{F}_{\boldsymbol{H}}\left( \boldsymbol{m,t} \right)$  $\boldsymbol{G}_{\boldsymbol{B}}\left( \boldsymbol{m,t} \right)\boldsymbol{=}\boldsymbol{K}_{\boldsymbol{D}}\boldsymbol{F}_{\boldsymbol{B}}\left( \boldsymbol{m,t} \right)$ | g year^-1^ | (M9)  (M10)  (M11) |
| Flux terms from death included  Predation mortality:  $\boldsymbol{D}_{\boldsymbol{iP}}\left( \boldsymbol{m,t} \right)\boldsymbol{=}\boldsymbol{\omega}_{\boldsymbol{i}}\boldsymbol{A}_{\boldsymbol{P}}\boldsymbol{V}_{\boldsymbol{P}}\int\boldsymbol{m}^{\boldsymbol{'}\boldsymbol{\alpha}_{\boldsymbol{P}}}\boldsymbol{\varphi}\left( \boldsymbol{m}^{\boldsymbol{'}}\boldsymbol{/m} \right)\boldsymbol{N}_{\boldsymbol{P}}\left( \boldsymbol{m}^{\boldsymbol{'}}\boldsymbol{,t} \right)\boldsymbol{d}\boldsymbol{m}^{\boldsymbol{'}}$  Intrinsic and senescence mortality  $\boldsymbol{D}_{\boldsymbol{iO}}\left( \boldsymbol{m} \right)\boldsymbol{=}\boldsymbol{\mu}\boldsymbol{m}^{\boldsymbol{-0.25}}\boldsymbol{+}\boldsymbol{k}_{\boldsymbol{s}}\boldsymbol{(m/}\boldsymbol{m}_{\boldsymbol{s}}\boldsymbol{)}^{\boldsymbol{p}_{\boldsymbol{s}}}$ | year^-1^ | (M12)  (M13) |
| Resulting in overall death rates:  $\boldsymbol{D}_{\boldsymbol{i}}\left( \boldsymbol{m,t} \right)\boldsymbol{=}\boldsymbol{D}_{\boldsymbol{iP}}\left( \boldsymbol{m,t} \right)\boldsymbol{+}\boldsymbol{D}_{\boldsymbol{iO}}\boldsymbol{(m)}$ | year^-1^ | (M14) |
| Flux in detritus from feeding, egestion and death:  $\boldsymbol{I}_{\boldsymbol{D}}\left( \boldsymbol{t} \right)\boldsymbol{=S}\int\boldsymbol{N}_{\boldsymbol{P}}\left( \boldsymbol{m,t} \right)\boldsymbol{E}_{\boldsymbol{P}}\boldsymbol{F}_{\boldsymbol{Pi}}\left( \boldsymbol{m,t} \right)\boldsymbol{+}{\boldsymbol{N}_{\boldsymbol{H}}\left( \boldsymbol{m,t} \right)\boldsymbol{E}_{\boldsymbol{H}}\boldsymbol{F}_{\boldsymbol{H}}\left( \boldsymbol{m,t} \right)\boldsymbol{+N}}_{\boldsymbol{B}}\left( \boldsymbol{m,t} \right)\boldsymbol{E}_{\boldsymbol{B}}\boldsymbol{F}_{\boldsymbol{B}}\boldsymbol{(m,t)}$  $\boldsymbol{+ S}\int\boldsymbol{N}_{\boldsymbol{P}}\left( \boldsymbol{m,t} \right)\boldsymbol{D}_{\boldsymbol{iO}}\left( \boldsymbol{m,t} \right)\boldsymbol{dm}$  $\boldsymbol{+ S}\int\boldsymbol{N}_{\boldsymbol{H}}\left( \boldsymbol{m,t} \right)\boldsymbol{D}_{\boldsymbol{iO}}\left( \boldsymbol{m,t} \right)\boldsymbol{dm}$  $\boldsymbol{+}\int\boldsymbol{N}_{\boldsymbol{B}}\left( \boldsymbol{m,t} \right)\boldsymbol{D}_{\boldsymbol{iO}}\left( \boldsymbol{m,t} \right)\boldsymbol{dm}$  $\boldsymbol{O}_{\boldsymbol{D}}\left( \boldsymbol{t} \right)\boldsymbol{=}\int\boldsymbol{N}_{\boldsymbol{B}}\left( \boldsymbol{m,t} \right)\boldsymbol{F}_{\boldsymbol{B}}\left( \boldsymbol{m,t} \right)\boldsymbol{dm}$ | gm^-2^ year^-1^  gm^-2^ year^-1^ | (M15)  (M16) |
| Flux in algal turfs from growth and grazing:  $\boldsymbol{I}_{\boldsymbol{T}}\left( \boldsymbol{t} \right)\boldsymbol{=alr}$  $\boldsymbol{O}_{\boldsymbol{T}}\left( \boldsymbol{t} \right)\boldsymbol{=}\int\boldsymbol{N}_{\boldsymbol{H}}\left( \boldsymbol{m,t} \right)\boldsymbol{F}_{\boldsymbol{H}}\left( \boldsymbol{m,t} \right)\boldsymbol{dm}$ | gm^-2^ year^-1^  gm^-2^ year^-1^ | (M17)  (M18) |
