# Supplementary material for: Mangroves reduce the vulnerability of coral reef fisheries to habitat degradation
Source: PLoS Biol. 2019 Nov 12;17(11):e3000510. doi: 10.1371/journal.pbio.3000510 (PMC6850520; doi:10.1371/journal.pbio.3000510)
Supplement: S2 Table — (DOCX) [file pbio.3000510.s002.docx]

**S2 Table.** Parameter definitions, values and units. *Refer to text for parameter explanation and justification

| Symbol | Definition | Value | Unit |
| --- | --- | --- | --- |
| 1. $\boldsymbol{[}\boldsymbol{m}_{\boldsymbol{min}}\boldsymbol{,}\boldsymbol{m}_{\boldsymbol{Pmin}}\boldsymbol{]}$ 2. $\boldsymbol{[}\boldsymbol{m}_{\boldsymbol{Pmin}}\boldsymbol{,}\boldsymbol{m}_{\boldsymbol{max}}\boldsymbol{]}$ 3. $\boldsymbol{[}\boldsymbol{m}_{\boldsymbol{Hmin}}\boldsymbol{,}\boldsymbol{m}_{\boldsymbol{max}}\boldsymbol{]}$ 4. $\boldsymbol{[}\boldsymbol{m}_{\boldsymbol{Bmin}}\boldsymbol{,}\boldsymbol{m}_{\boldsymbol{max}}\boldsymbol{]}$ | Body mass ranges for:   1. Plankton 2. Predatory fish 3. Herbivorous fish 4. Benthic detritivores | 1. 10^-12^,10^-1.5^ 2. 10^-1.5^,10^3.5^ 3. 10^-1.5^,10^3.5^ 4. 10^-4^,10^3.5^ | g |
| $\boldsymbol{alr}$ | Flux in turf algae | 110 | gm^-2^ year^-1^ |
| $\boldsymbol{\omega}$ | Fraction of time spent by predators feeding in the predator, herbivore or invertebrate spectrum | $\omega_{P}$ = 0.33  $\omega_{H}$ = 0.17  $\omega_{B}$ = 0. 5 |  |
| $\boldsymbol{\beta}$ | Log of modal predator-prey mass ratio (PPMR) | Log(100) |  |
| $\boldsymbol{ϭ}$ | Measure of the width of the log (PPMR) distribution | 1.0 |  |
| $\boldsymbol{A}$ | Volume of water required to meet metabolic demands by a unit mass, $m$ | $A_{P}$: 6.4  $A_{H}$: 0.2  $A_{B}$: 0.1 | m^2^ year^-1^ |
| $\boldsymbol{\alpha}$ | Exponent of mass in volume of water required | $\alpha_{P,}{\alpha_{H}, \alpha}_{B}$: 0.75 |  |
| $\boldsymbol{S}$ | Proportion of dead and egested material that reaches the reef and becomes detritus | 0.8 |  |
| $\boldsymbol{E}$ | Fraction of food egested | $E_{P},{E_{H,}E}_{B}$: 0.4, |  |
| $\boldsymbol{K}$ | Gross growth conversion efficiency. | $K_{P},{K_{H},K}_{B}$: 0.15  $K_{T}$,$K_{D}$: 0.1 |  |
| $\boldsymbol{\mu}$ | Residual natural mortality | 0.2 |  |
| $\boldsymbol{m}_{\boldsymbol{s}}$ | Log body mass size at senescence | 3 |  |
| $\boldsymbol{k}_{\boldsymbol{s}}$ | Constant for senescence mortality | 0.1 |  |
| $\boldsymbol{p}_{\boldsymbol{s}}$ | Exponent of senescence curve | 0.3 |  |
