# Supplementary material for: Mangroves reduce the vulnerability of coral reef fisheries to habitat degradation
Source: PLoS Biol. 2019 Nov 12;17(11):e3000510. doi: 10.1371/journal.pbio.3000510 (PMC6850520; doi:10.1371/journal.pbio.3000510)
Supplement: S3 Table — (DOCX) [file pbio.3000510.s003.docx]

**S3 Table.** Allocation of empirically measured crevice densities to model fish body mass increments based on length-weight conversion parameters; a = 0.025, b = 3 and the relationship: weight = a * length^ b. Orange squares depict which body mass increments a given empirical refuge density were assigned to.

| Log10 body mass increment | Equivalent size (cm) | Fine branching corals (<2.5cm) | Medium branching corals (<5cm) | Crevice 0-5cm | Crevice 5-10cm | Crevice 10-15cm | Crevice 15-20cm | Crevice 20-25cm | Crevice 25-30cm | Crevice 30-35cm | Crevice 35-40cm | Crevice 40-45cm | Crevice 45-50cm |
| --- | --- | --- | --- | --- | --- | --- | --- | --- | --- | --- | --- | --- | --- |
| -1.5 | 1.1 |  |  |  |  |  |  |  |  |  |  |  |  |
| -1.4 | 1.2 |  |  |  |  |  |  |  |  |  |  |  |  |
| -1.3 | 1.3 |  |  |  |  |  |  |  |  |  |  |  |  |
| -1.2 | 1.4 |  |  |  |  |  |  |  |  |  |  |  |  |
| -1.1 | 1.5 |  |  |  |  |  |  |  |  |  |  |  |  |
| -1.0 | 1.6 |  |  |  |  |  |  |  |  |  |  |  |  |
| -0.9 | 1.7 |  |  |  |  |  |  |  |  |  |  |  |  |
| -0.8 | 1.9 |  |  |  |  |  |  |  |  |  |  |  |  |
| -0.7 | 2.0 |  |  |  |  |  |  |  |  |  |  |  |  |
| -0.6 | 2.2 |  |  |  |  |  |  |  |  |  |  |  |  |
| -0.5 | 2.3 |  |  |  |  |  |  |  |  |  |  |  |  |
| -0.4 | 2.5 |  |  |  |  |  |  |  |  |  |  |  |  |
| -0.3 | 2.7 |  |  |  |  |  |  |  |  |  |  |  |  |
| -0.2 | 2.9 |  |  |  |  |  |  |  |  |  |  |  |  |
| -0.1 | 3.2 |  |  |  |  |  |  |  |  |  |  |  |  |
| 0.0 | 3.4 |  |  |  |  |  |  |  |  |  |  |  |  |
| 0.1 | 3.7 |  |  |  |  |  |  |  |  |  |  |  |  |
| 0.2 | 4.0 |  |  |  |  |  |  |  |  |  |  |  |  |
| 0.3 | 4.3 |  |  |  |  |  |  |  |  |  |  |  |  |
| 0.4 | 4.6 |  |  |  |  |  |  |  |  |  |  |  |  |
| 0.5 | 5.0 |  |  |  |  |  |  |  |  |  |  |  |  |
| 0.6 | 5.4 |  |  |  |  |  |  |  |  |  |  |  |  |
| 0.7 | 5.9 |  |  |  |  |  |  |  |  |  |  |  |  |
| 0.8 | 6.3 |  |  |  |  |  |  |  |  |  |  |  |  |
| 0.9 | 6.8 |  |  |  |  |  |  |  |  |  |  |  |  |
| 1.0 | 7.4 |  |  |  |  |  |  |  |  |  |  |  |  |
| 1.1 | 8.0 |  |  |  |  |  |  |  |  |  |  |  |  |
| 1.2 | 8.6 |  |  |  |  |  |  |  |  |  |  |  |  |
| 1.3 | 9.3 |  |  |  |  |  |  |  |  |  |  |  |  |
| 1.4 | 10 |  |  |  |  |  |  |  |  |  |  |  |  |
| 1.5 | 11 |  |  |  |  |  |  |  |  |  |  |  |  |
| 1.6 | 12 |  |  |  |  |  |  |  |  |  |  |  |  |
| 1.7 | 13 |  |  |  |  |  |  |  |  |  |  |  |  |
| 1.8 | 14 |  |  |  |  |  |  |  |  |  |  |  |  |
| 1.9 | 15 |  |  |  |  |  |  |  |  |  |  |  |  |
| 2.0 | 16 |  |  |  |  |  |  |  |  |  |  |  |  |
| 2.1 | 17 |  |  |  |  |  |  |  |  |  |  |  |  |
| 2.2 | 19 |  |  |  |  |  |  |  |  |  |  |  |  |
| 2.3 | 20 |  |  |  |  |  |  |  |  |  |  |  |  |
| 2.4 | 22 |  |  |  |  |  |  |  |  |  |  |  |  |
| 2.5 | 23 |  |  |  |  |  |  |  |  |  |  |  |  |
| 2.6 | 25 |  |  |  |  |  |  |  |  |  |  |  |  |
| 2.7 | 27 |  |  |  |  |  |  |  |  |  |  |  |  |
| 2.8 | 29 |  |  |  |  |  |  |  |  |  |  |  |  |
| 2.9 | 32 |  |  |  |  |  |  |  |  |  |  |  |  |
| 3.0 | 34 |  |  |  |  |  |  |  |  |  |  |  |  |
| 3.1 | 37 |  |  |  |  |  |  |  |  |  |  |  |  |
| 3.2 | 40 |  |  |  |  |  |  |  |  |  |  |  |  |
| 3.3 | 43 |  |  |  |  |  |  |  |  |  |  |  |  |
| 3.4 | 47 |  |  |  |  |  |  |  |  |  |  |  |  |
| 3.5 | 50 |  |  |  |  |  |  |  |  |  |  |  |  |
