# Supplementary material for: Mangroves reduce the vulnerability of coral reef fisheries to habitat degradation
Source: PLoS Biol. 2019 Nov 12;17(11):e3000510. doi: 10.1371/journal.pbio.3000510 (PMC6850520; doi:10.1371/journal.pbio.3000510)
Supplement: S4 Table — (DOCX) [file pbio.3000510.s004.docx]

**S4 Table.** Supplemental predator and herbivore fish densities from 16 mangrove sites in Belize

| Mangrove site | Predator density (m^2^) | Herbivore density (m^2^) |
| --- | --- | --- |
| 1 | 1.61 | 0.09 |
| 2 | 0.59 | 0.30 |
| 3 | 1.62 | 0.13 |
| 4 | 0.67 | 0.09 |
| 5 | 0.59 | 0.15 |
| 6 | 0.82 | 0.15 |
| 7 | 0.43 | 0.06 |
| 8 | 1.29 | 0.10 |
| 9 | 0.56 | 0.04 |
| 10 | 3.31 | 0.15 |
| 11 | 0.33 | 0.20 |
| 12 | 0.63 | 0.03 |
| 13 | 0.74 | 0.09 |
| 14 | 0.19 | 0.03 |
| 15 | 0.85 | 0.03 |
| 16 | 1.24 | 0.08 |

**S1 Text: Model descriptions and parameter justification**

This study uses the ecosystem model (previously food web) described in Rogers, Blanchard [1], with modifications relating to the vulnerability function and algal-herbivore dynamics. A number of model parameters have been updated in line with new or improved information. In addition, model inputs and outputs are expressed in square, rather than cubic meters, allowing us to directly use and compare fish and benthic data from coral reef surveys, expressed in square meters. When necessary, we assumed an average coral reef depth of 10m to transform parameters. This section provides details of the model structure and functions to accompany model equations presented in S1 Table, along with justifications for model parameters shown in S2 Table.

*Fish recruitment and planktonic primary production*

Coupling in the model consists of predation and production linkages between three size-structured assemblages: predatory fish, herbivorous fish and benthic invertebrates. In each assemblage, we are concerned with the continuous function *N* (*m*, *t*) (m^-2^ g^-1^) which gives the density per unit mass per unit area for organisms of mass *m* at time *t*. The continuous processes of growth *G* and mortality *D* that arise from organisms encountering and eating available and suitable food govern the temporal dynamics and lead to partial differential equations for each size spectrum *i* $\in$(P=pelagic predators, H=herbivorous fish, B =benthic detritivores) (See S1 Table, M1,M2,M3).

Renewal of the abundance / density of predatory and herbivorous fish in the smallest body size is determined by a fixed intercept, assuming constant recruitment. Empirical measures of recruitment for whole reef fish assemblages are lacking and can be highly variable among species, both temporally and spatially [2]. We chose an intercept value that yielded approximately ten individuals per square meter for each fish guild. The value approximated reported densities of small (< 4cm) reef fish on the Great Barrier Reef and in the Caribbean, from studies that used methods revealing both cryptic and non-cryptic species surveys [~ 12 - 16 pe square metre; 3, 4]. The minimum body size () for reef fish was also set based on these surveys.

The planktonic resource (and implicitly, the larval stages of reef fish) that provides food for predators in the smallest body size is held as a constant function of body mass through time *N_pp_*=*c*·*m*^-1^. We set the intercept for plankton at ten times that of reef fish recruits to roughly account for losses associated with energy transfer from plankton to reef fish recruits as in Woodworth et al [5]. Chosen value yields biomass densities of herbivorous fish that are comparable to those observed on un-fished reefs in Bonaire (see main text Fig 2).

*Supplemental recruitment from mangrove nurseries*

For this study, half of our modelled scenarios were associated with a mangrove nursery effect, whereby supplemental fish were added to both the predatory and herbivorous fish spectra. The density (m^2^) of additional fish was determined by field measurements within mangrove fringes at 16 locations in the Yucatan and Belize, Central America (for full details see[6]). In these studies, surveyed fish were designated as either predators (feeding on other fish and / invertebrates) or herbivores, resulting in two values for each of the 16 sites (S4 Table). At each mangrove site, ten 20 m × 2 m transects were laid along the fringe and all fishes quantified visually with size estimated to the nearest centimetre.

It was then necessary to find a relevant scaling function for the flux of fish leaving mangroves and reaching the reef. To do this we compared the areas of reef and mangrove fringe habitats. A complicating factor is that fish use the mangrove fringe which scales with perimeter rather than total forest area. Estimates of perimeter are highly sensitive to the scale of measurement since most natural habitat boundaries have a fractal – or at least partly fractal – structure[7, 8]. The original estimates of mangrove perimeter, reported in[6], were based on Landsat TM satellite imagery at a 30 m resolution. This is adequate for comparative analyses but not ideal for estimating the magnitude of perimeter at ecologically relevant scales. We therefore calculated a scaling relationship to a more reasonable 1 m resolution. This was done for the same mangrove habitat (fringing *Rhizophora*) in the Turks and Caicos, which was a test site for a series of mangrove remote sensing comparisons and where images of multiple resolutions were acquired almost simultaneously and ground truthed[9, 10]. The perimeter of the same mangrove fringe estimated using imagery with 1 m pixels was 12 times greater than that when the imagery had a coarse resolution of 30 m. Assuming a functional width of mangrove fringe for reef fishes of 3 m (PJ Mumby, pers. obs.), we then re-estimated the area of mangrove fringing habitat in Belize and found it to be double that of the adjacent forereef.

To estimate the flux of fish migrating from mangroves to reefs we therefore doubled the observed density to account for the relatively large area of mangrove habitat. To capture the realities of ontogenetic habitat migration, supplemental fish were added to the reef model at a size of ~16cm^11^ (log10 body mass increment 2 in the model). As a result, at each time step in the model, the density of fish in the log10 body mass increment 2, was inflated by adding the supplemental density migrating from mangroves, to the current density at that time. Predation, predation vulnerability and grazing functions were then applied as normal to modified size spectra.

*Model comparison from Bonaire*

To test whether the model can capture the relationship between habitat structure and fish biomass and size spectra, we compared field and model data on herbivores from Bonaire, southern Caribbean[11]. Bonaire was an appropriate place to undertake this assessment because there is no herbivore fishery[12] and the mangrove impacts are likely negligible because they are so far from the reefs surveyed. Reef habitats were sampled along an explicit gradient of habitat complexity[13]. One hundred 25 m^2^ plots were sampled across five levels of habitat complexity within the same geomorphological reef zone (*Orbicella* reef). Herbivorous fishes were quantified to species and size estimated to the nearest centimetre. The distribution of reef gaps / holes were also surveyed and used to describe the prey refuge function in the model.

*Empirical comparison between model and field data*

To explore congruence between model predictions and empirical fish assemblage structure we re-examined data from an earlier study that explicitly stratified sampling across six reef systems half of which lacked mangrove nurseries and were isolated from a mangrove source[6]. A total of 23 sites were sampled with a minimum of three per system (atoll or section of the Mesoamerican barrier reef). All field data were taken on the outer forereef at a depth of ca 10 m and across a modest range of habitat complexity within the same geomorphological zone (*Orbicella* reef). The biomass of all carnivorous species was quantified for each site. Fish surveys utilised visual census with size attempted to the nearest centimetre. Sample unit size and number was scaled to the habitat and behaviour of the fish. The transect dimensions and numbers (given in parentheses) at each site were 30 m × 2m (6) for smaller benthic species; 30 m × 4m (10) for scarids, acanthurids, pomacanthids, diodontids and monacanthids; 100 m × 4m (6) for haemulids, chaetodontids, small serranids and labrids; and 100 m × 6m (6) for lutjanids, carangids, planktivorous labrids, large serranids and other large predators. Lengths were converted to biomass using standard allometric relationships[14]. Rugosity was sampled using a fine chain transect of 2 m length (n=10 per site).

*Predation*

The feeding rate *F_Pi_* (*m*,*t*) of a given size predator is a function of the preference for prey $\omega_{i}$in spectrum *i*, the area of reef searched per unit time (as a function of its body size), $A_{P}m^{\alpha_{P}}$(m^-2^yr^-1^), the proportion of prey vulnerable to predation (not inhabiting refugia in the reef structure) $V(m)$ in spectrum *i,* and the available prey that are suitably sized food in spectrum *i*$, \int\varphi\left( m/{m^{'}} \right)N_{i}(m^{'},t)m^{'}dm^{'}$ (See Table S1, M6).

The parameter value for the area of reef searched by predatory fish is chosen based on volume of water searched in previous size-structured food web models, corrected for a change from m^3^ to m^2^ (Blanchard 2012). In addition, the value falls within the range of estimates for the intercept of allometric home range size of predatory reef fish from a recent review [15].

The vulnerability of prey fish of size *m’* to predation at time *t,* is given by the vulnerability function

$$V_{i}\left( m',t \right)=1-\frac{refuge (m)}{N_{P}\left( m,t \right)+ N_{H}\left( m,t \right)}$$

where *refuge (m)* is the density of available reef crevices that are suitably sized for the body mass increment of fish (see Table S3), determined from field studies, and therefore varying between reefs. With this function, the vulnerability of a given size prey can change through time in response to changes in the abundance of equal-sized predator and herbivore competitors.

Given that the majority of epifaunal invertebrates on coral reefs inhabit and use macroalgal canopies as refugia [16] we assume that the spatial scale of structural complexity inferred by reef-building corals will have little direct impact on the vulnerability of benthic invertebrates to predation. Furthermore, our model captures refuge availability and its consequences above a 2.5cm minimum body size which is much larger than the majority of reef invertebrates. Subsequently, we set the vulnerability of the benthic invertebrate spectrum to one for all reef scenarios and reef types explored with the model.

The probability of a predator of size *m* eating an encountered prey of size $m'$is given by the lognormal probability density function:

$$\varphi\left( m,m^{'} \right)=e\left[ \frac{-{(\log\left( m/m' \right)-\beta)}^{2}}{2\sigma^{2}} \right].\frac{1}{(\sigma\sqrt{2\pi)}}$$

when $m$ > $m'$ and $\varphi\left( m,m^{'} \right)=0$ otherwise, where $\beta$ is the logarithm of the preferred predator: prey mass ratio and $\sigma$ the width.

*Herbivory*

Herbivorous fish compete within their size spectra for algal turfs. The feeding rate of a given size herbivore *F_H_* (*m*,*t*) depends on the area of reef searched per unit time $A_{H}m^{\alpha_{H}}$ (m^-2^yr^-1^) and the available biomass density of algal turfs (see Table S1, M17 and M18). The area of reef searched is determined by the intercept of allometric home range sizes for herbivorous fish, from visual surveys [15], and the rate of algal turf production, *alr* falls within a range of empirically derived rates of net primary production in the Caribbean [110 - 274; 17]. We chose a turf production value at the low end of this range, assuming that not all substrate on the reef would support algal turfs, and because it yielded herbivore growth rates and densities comparable with un-fished reefs in Bonaire (see main text Figure 4).

*Invertebrates and detritus*

Like herbivores, benthic invertebrates compete within their size spectra for an unstructured pool of resources, in this case detritus. The feeding rate of a given size invertebrate *F_B_* (*m*,*t*) depends on the area of reef either searched per unit time $A_{B}m^{\alpha_{B}}$ (m^-2^yr^-1^) and the available biomass density of detritus (see Table S1, M15 and M16). In the absence of any empirical data, the area of reef searched is half that of herbivorous fish, implicitly taking into account their smaller body size. Detritus enters the system as a result of death and defecation from each of the size spectra and a sinking rate, which determines how much material is recycled back onto the reef. In this iteration of the model, the sinking rate for detritus is set at 0.8, based on studies that have showed that most organic material is recycled in the shallow water systems of coral reefs [18].

The intercept and slope parameters for benthic invertebrates have been up-dated in this iteration of the model in light of a recent study providing information on biomass density and productivity of benthic crustaceans in different coral reef habitats [19].

**Supplementary References**

1. Rogers A, Blanchard Julia L, Mumby Peter J. Vulnerability of coral reef fisheries to a loss of structural complexity. Curr Biol. 2014;24(9):1000-5. doi: <http://dx.doi.org/10.1016/j.cub.2014.03.026>.

2. Doherty PJ, Williams DM. The replenishment of coral reef fish populations. Oceanography and Marine Biology: An Annual Review. 1988;26(48):551. PubMed PMID: WOS:A1988Q990100008.

3. Ackerman JL, Bellwood DR. Reef fish assemblages: a re-evaluation using enclosed rotenone stations. Mar Ecol-Prog Ser. 2000;206:227-37.

4. Depczynski M, Fulton C, Marnane M, Bellwood D. Life history patterns shape energy allocation among fishes on coral reefs. Oecologia. 2007;153(1):111-20. doi: 10.1007/s00442-007-0714-2.

5. Woodworth-Jefcoats PA, Polovina JJ, Dunne JP, Blanchard JL. Ecosystem size structure response to 21st century climate projection: large fish abundance decreases in the central North Pacific and increases in the California Current. Global Change Biology. 2013;19(3):724-33. doi: 10.1111/gcb.12076.

6. Mumby PJ, Edwards AJ, Arias-Gonzalez JE, Lindeman KC, Blackwell PG, Gall A, et al. Mangroves enhance the biomass of coral reef fish communities in the Caribbean. Nature. 2004;427(6974):533-6. PubMed PMID: ISI:000188721800039.

7. Farina A. Principles and methods in Landscape Ecology. London: Chapman and Hall; 1998. 235 p.

8. Hastings HM, Pekelney R, Monticciolo R, Vun Kannon D, Del Monte D. Time Scales, Persistence and Patchiness. BioSyst. 1982;15(4):281-9. PubMed PMID: ISI:A1982QD41400002.

9. Green EP, Mumby PJ, Edwards AJ, Clark CD, Ellis AC. The assessment of mangrove areas using high resolution multispectral airborne imagery. J Coast Res. 1998;14(2):433-43. PubMed PMID: ISI:000073189300005.

10. Green EP, Clark CD, Mumby PJ, Edwards AJ, Ellis AC. Remote sensing techniques for mangrove mapping. Int J Remote Sens. 1998;19(5):935-56. PubMed PMID: ISI:000073174700009.

11. Rogers A, Blanchard JL, Newman SP, Dryden C, Mumby PJ. High refuge availability on coral reefs increases the vulnerability of reef-associated predators to overexploitation. Ecology. 2018;99:450-63.

12. Steneck RS, Arnold SN, Boenish R, Leon Rd, Mumby PJ, Rasher DB, et al. Managing recovery resilience in coral reefs against climate-induced bleaching and hurricanes: A 15 year case study from Bonaire, Dutch Caribbean. Frontiers in Marine Science. 2019;6:265. doi: 10.3389/fmars.2019.00265.

13. Newman SP, Meesters EH, Dryden CS, Williams SM, Sanchez C, Mumby PJ, et al. Reef flattening effects on total richness and species responses in the Caribbean. The Journal of animal ecology. 2015. doi: 10.1111/1365-2656.12429. PubMed PMID: 26344713.

14. Bohnsack JA, Harper DE. Length-weight relationships of selected marine reef fishes from the southeastern United States and the Caribbean. Miami: National Fish & Wildlife Service, 1988 NOAA Technical Memorandum NMFS-SEFC-215.

15. Nash K, Welsh J, Graham NJ, Bellwood D. Home-range allometry in coral reef fishes: comparison to other vertebrates, methodological issues and management implications. Oecologia. 2015;177(1):73-83. doi: 10.1007/s00442-014-3152-y.

16. Roff G, Wabnitz CCC, Harborne AR, Mumby PJ. Macroalgal associations of motile epifaunal invertebrate communities on coral reefs. Marine Ecology. 2013;34(4):409-19. doi: 10.1111/maec.12040.

17. Opitz S. Trophic interactions in Caribbean coral reefs: International Center for Living Aquatic Resources Managament; 1996.

18. Hatcher BG. Coral reef primary productivity: A beggar's banquet. Trends in Ecology & Evolution. 1988;3(5):106-11. doi: <http://dx.doi.org/10.1016/0169-5347(88)90117-6>. PubMed PMID: 21227159.

19. Kramer MJ, Bellwood DR, Bellwood O. Benthic crustacea on coral reefs: a quantitative survey. Mar Ecol-Prog Ser. 2014;511:105-16. doi: 10.3354/meps10953.
